# Supplementary figures and images for: Autophagic stress activates distinct compensatory secretory pathways in neurons
Source: bioRxiv. 2024 Nov 7:2024.11.07.621551. Preprint. [Version 1] doi: 10.1101/2024.11.07.621551 (PMC11580983; doi:10.1101/2024.11.07.621551)

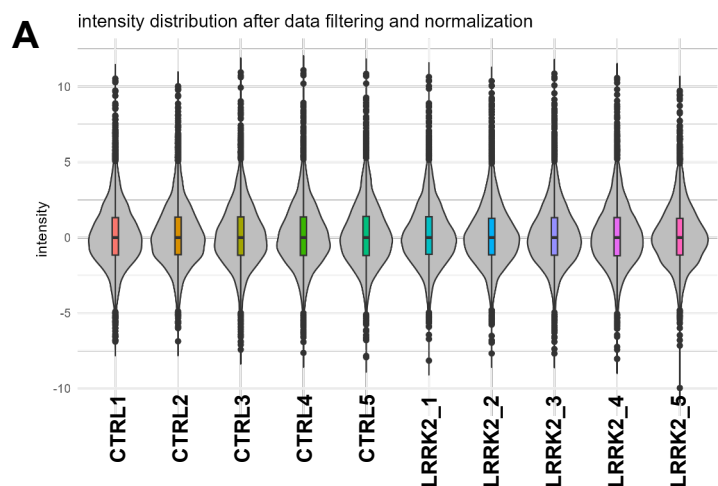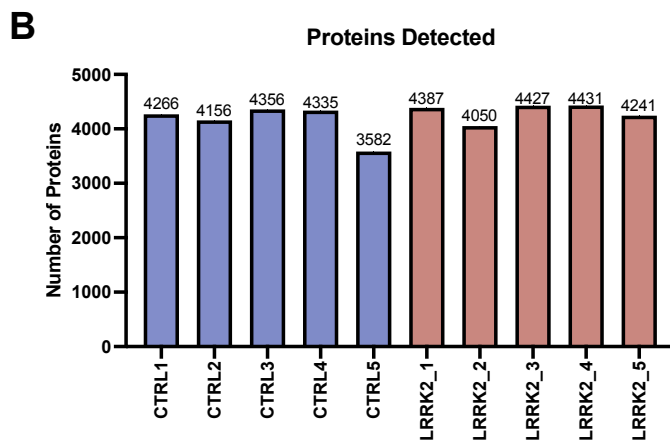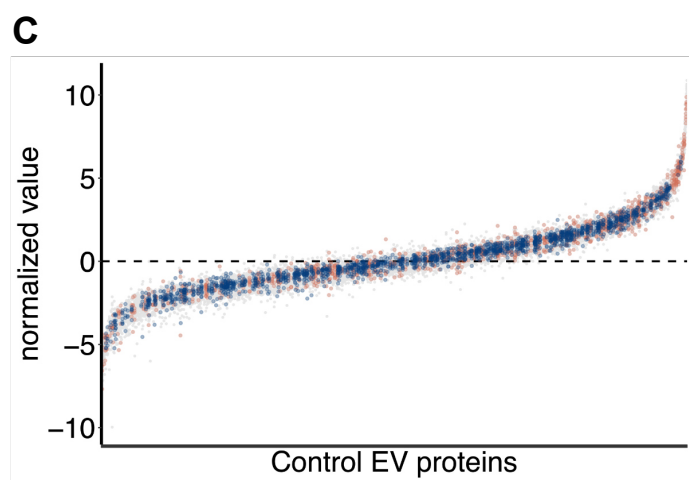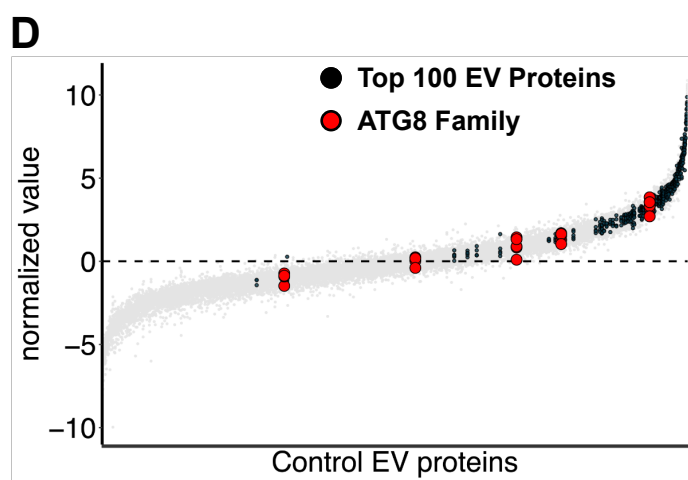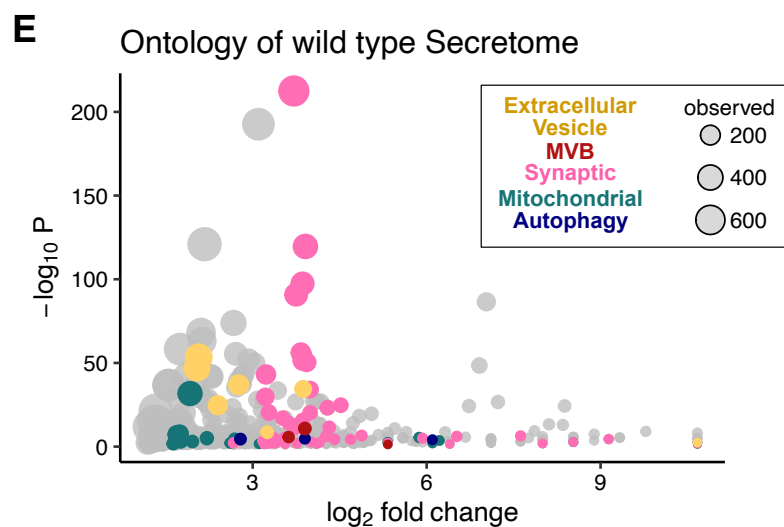

**A**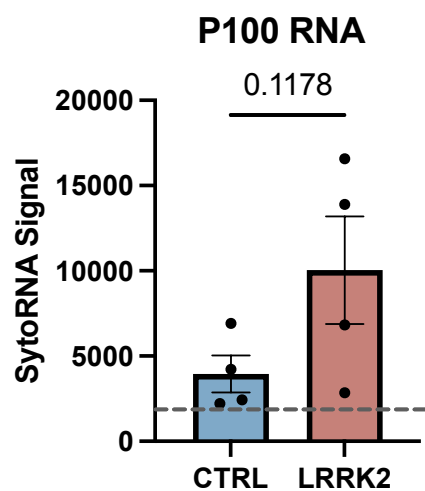**B****Top miRNAs by Sample**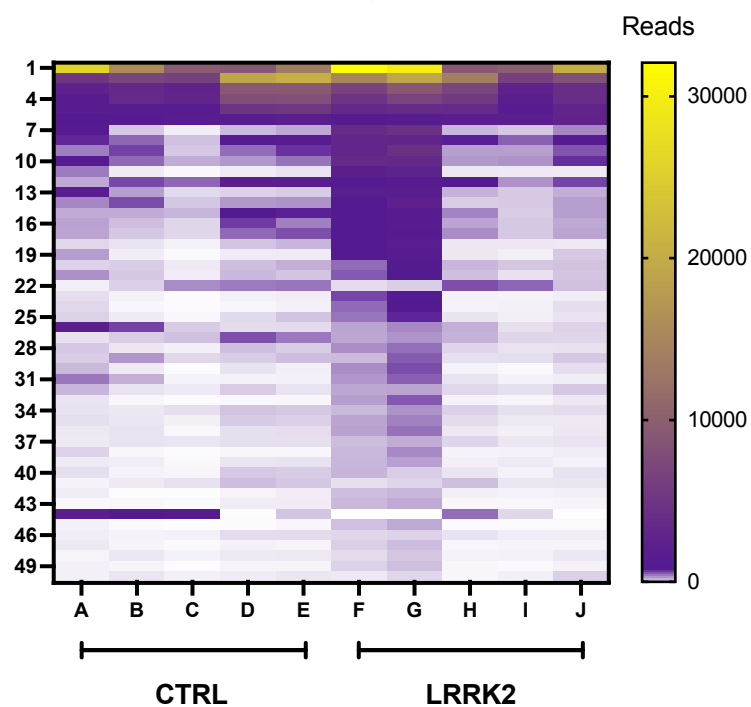**C****MA plot**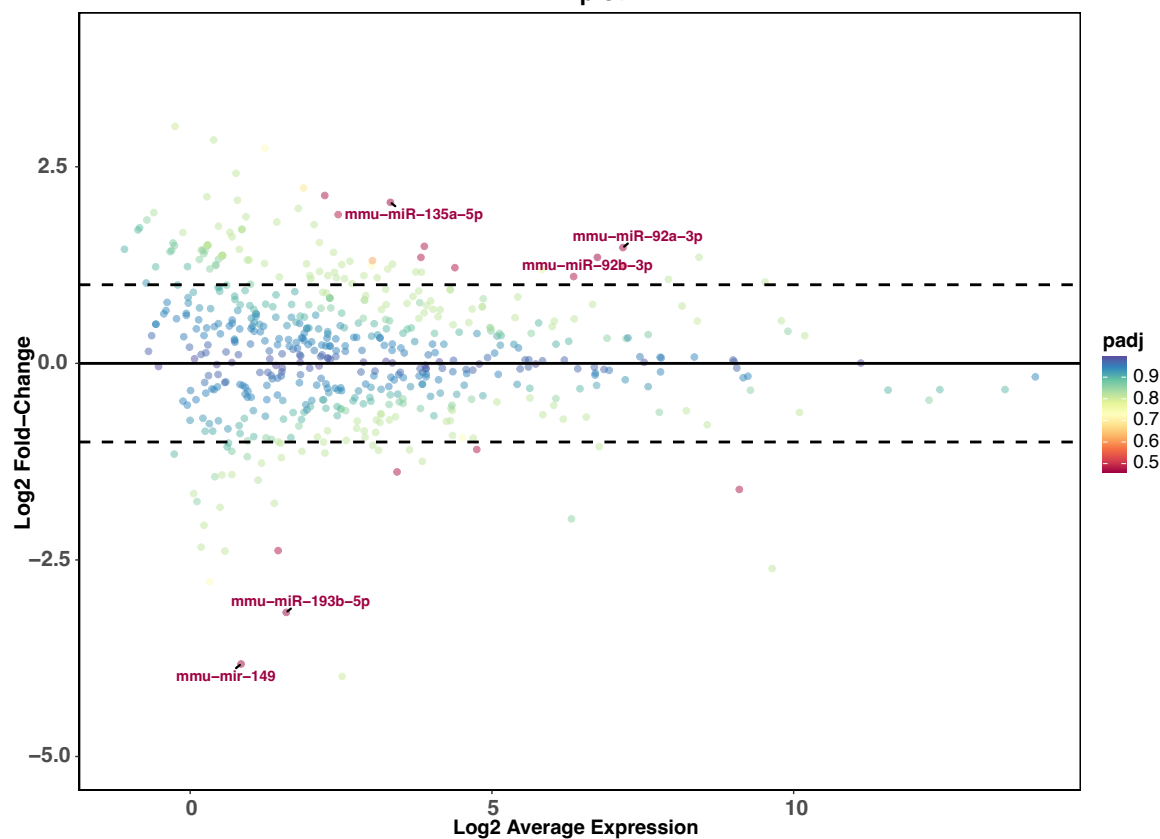

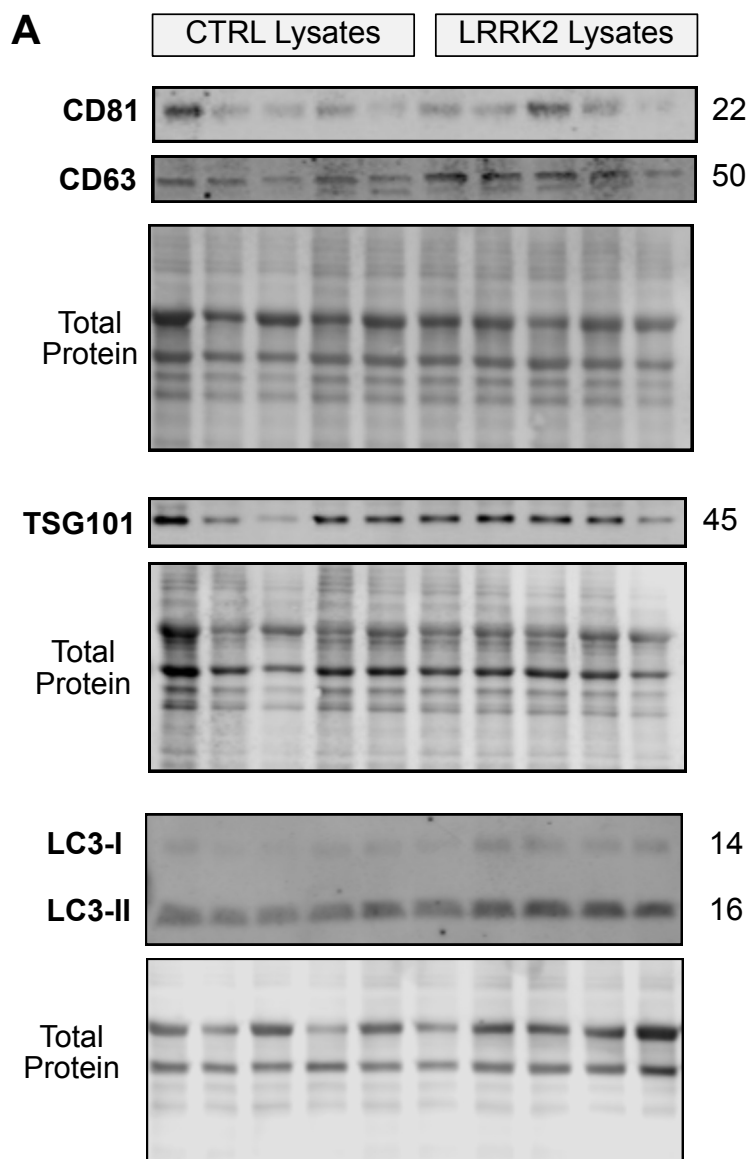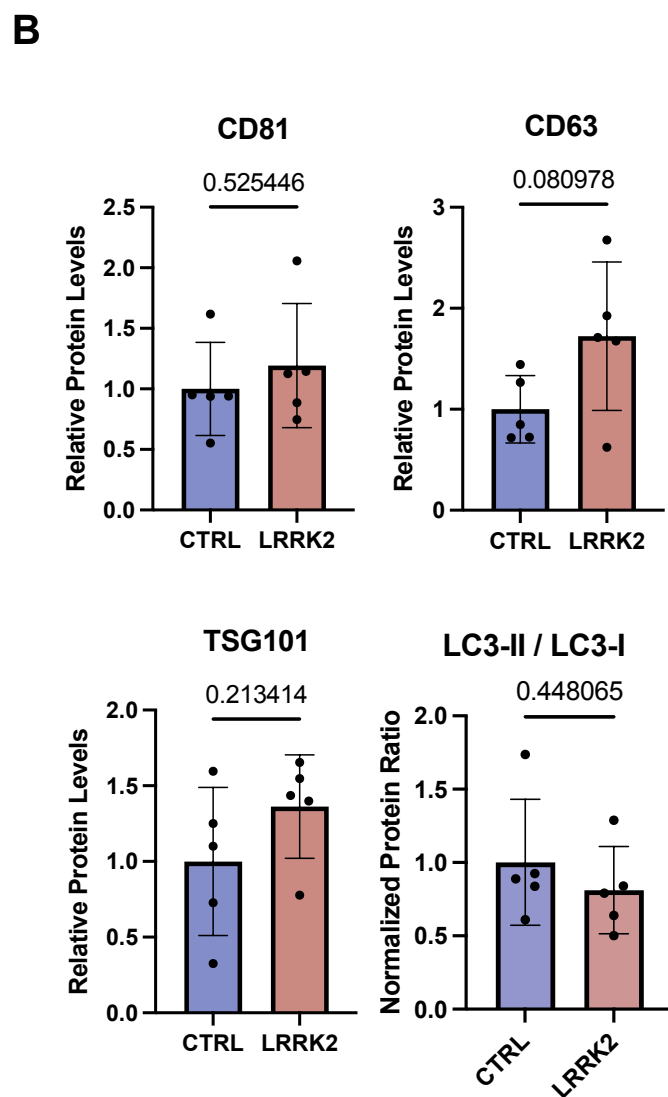

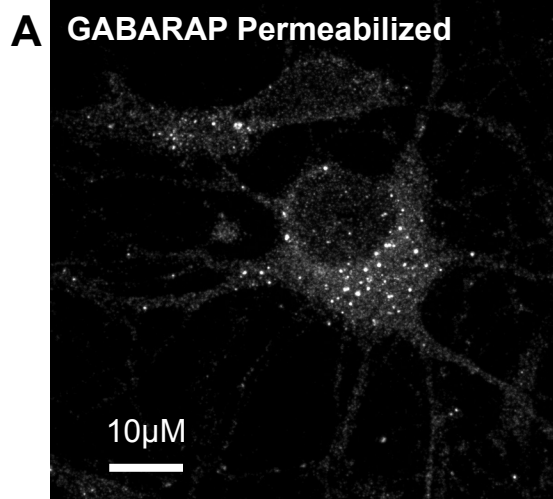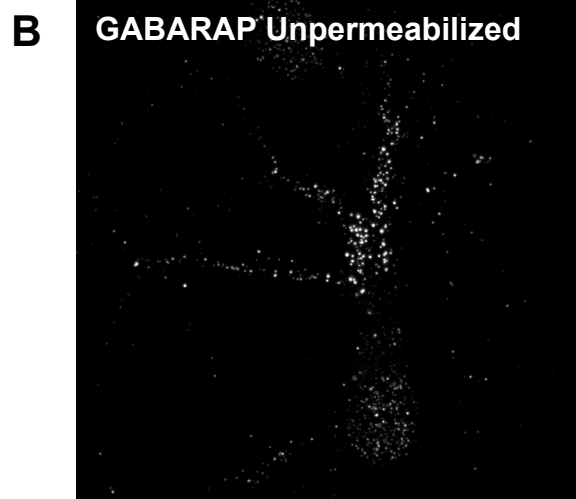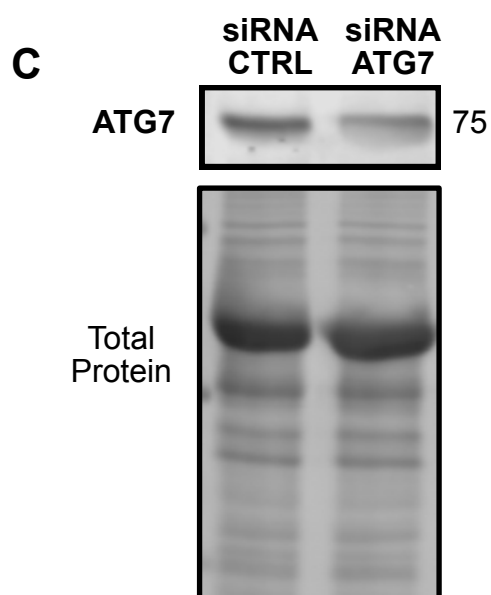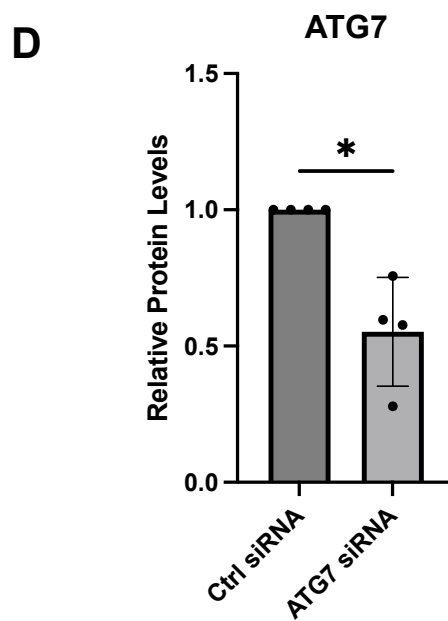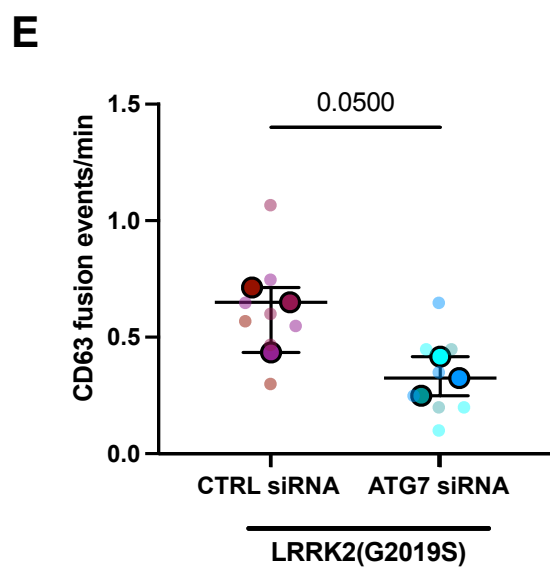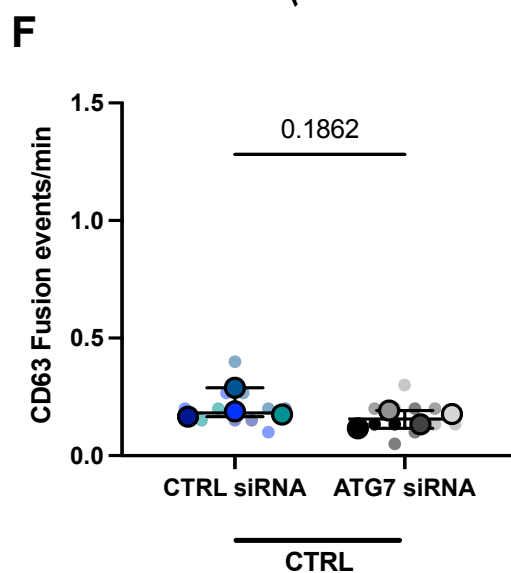

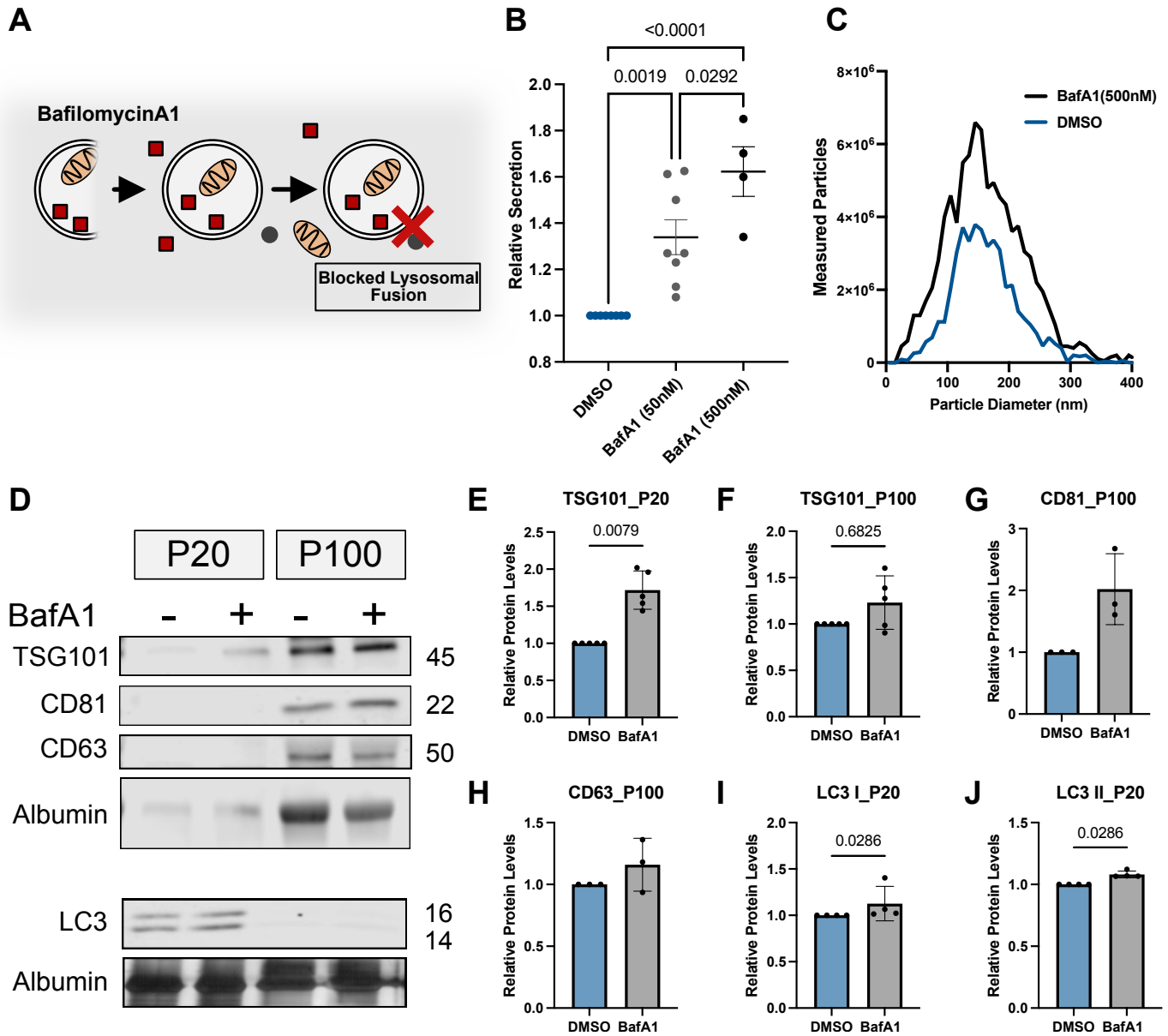

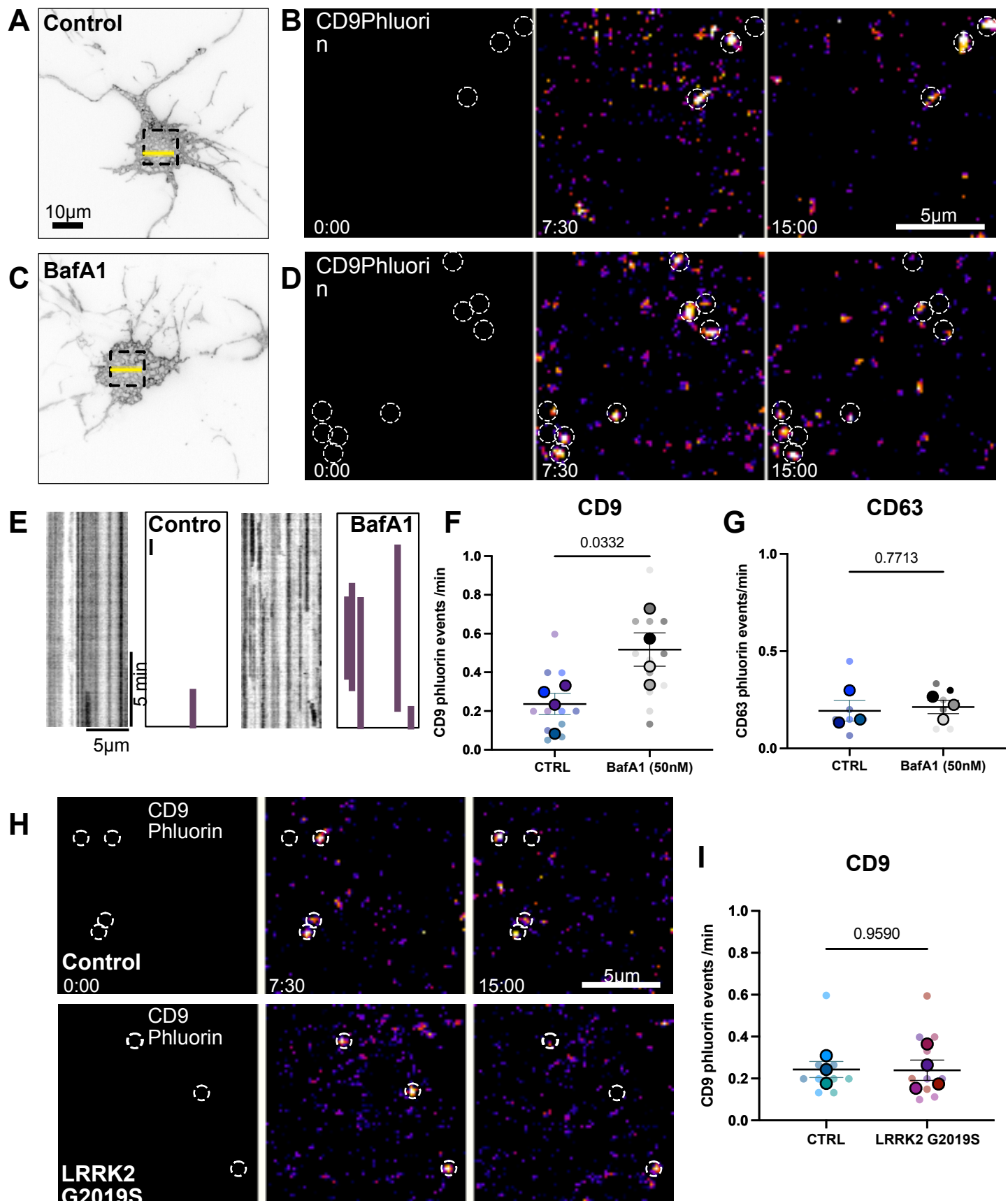

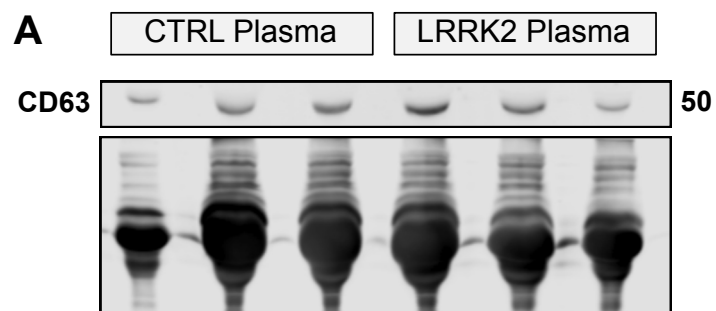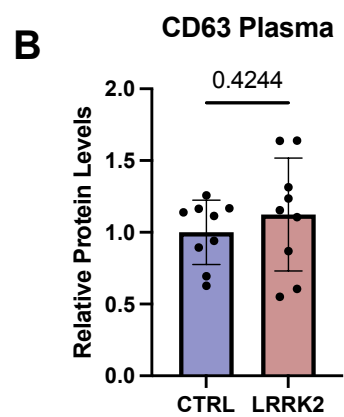

Supplement: Supplement 1 [file media-1.pdf]
